# Involvement of *HB-EGF/Ascl1/Lin28a* Genes in Dedifferentiation of Adult Mammalian Müller Glia

**DOI:** 10.3389/fmolb.2020.00200

**Published:** 2020-08-14

**Authors:** Megan L. Stanchfield, Sarah E. Webster, Mark K. Webster, Cindy L. Linn

**Affiliations:** Department of Biological Sciences, Western Michigan University, Kalamazoo, MI, United States

**Keywords:** mammalian retinal regeneration, Müller glia, dedifferentiation, retinal pigment epithelium, α7 nicotinic acetylcholine receptor agonist

## Abstract

Previous studies from this lab have determined that dedifferentiation of Müller glia occurs after eye drop application of an α7 nicotinic acetylcholine receptor (nAChR) agonist, PNU-282987, to the adult rodent eye. PNU-282987 acts on α7 nAChRs on retinal pigment epithelial cells to stimulate production of Müller-derived progenitor cells (MDPCs) and ultimately lead to neurogenesis. This current study was designed to test the hypothesis that the activation of genes involved in the *HB-EGF/Ascl1/Lin28a* signaling pathway in Müller glia leads to the genesis of MDPCs. RNA-seq was performed on a Müller glial cell line (rMC-1) following contact with supernatant collected from a retinal pigment epithelial (RPE) cell line treated with PNU-282987. Differentially regulated genes were compared with published literature of Müller glia dedifferentiation that occurs in lower vertebrate regeneration and early mammalian development. *HB-EGF* was significantly up-regulated by 8 h and expression increased through 12 h. By 48 h, up-regulation of *Ascl1* and *Lin28a* was observed, two genes known to be rapidly induced in dedifferentiating zebrafish Müller glia. Up-regulation of other genes known to be involved in mammalian development and zebrafish regeneration were also observed, as well as down-regulation of some factors necessary for Müller glia cell identity. RNA-seq results were verified using qRT-PCR. Using immunocytochemistry, the presence of markers associated with MDCP identity, *Otx2*, *Nestin*, and *Vsx2*, were found to be expressed in the 48 h treatment group cultures. This study is novel in its demonstration that Müller glia in adult rodents can be induced into regenerative activity by stimulating genes involved in the *HB-EGF/Ascl1/Lin28a* pathway that leads to MDPCs after introducing conditioned media from PNU-282987 treated RPE. This study furthers our understanding of the mechanism by which Müller glia dedifferentiate in response to PNU-282987 in the adult mammalian retina.

## Introduction

Neurodegenerative diseases of the retina such as glaucoma, macular degeneration, retinal ischemia, and diabetic retinopathy lead to irreversible loss of retinal cells in adult mammals (Velez-Montoya et al., 2013; Weinreb et al., 2014; Duh et al., 2017; Dattilo et al., 2018). However, within the mammalian neuronal retina, neurogenesis does not typically occur after early development (Dhomen et al., 2006; Lamba et al., 2006; Sehgal et al., 2008). Decades of research has been performed in an effort to understand the inability of mammals to regenerate the central nervous system in adults, given the fact that many other animals are able to do so throughout adulthood (Dmitrieva et al., 2007; Gorsuch and Hyde, 2014). It is thought that the mechanism by which central nervous system regeneration occurs exists within mammals but has been inhibited through evolutionary time (Chen and Tonegawa, 1998; Nicholls et al., 1999; Tanaka and Ferretti, 2009; Echeverri, 2020).

Previous work from this lab has shown that administration of PNU-282987, an α7 nAChR-specific agonist (Bodnar et al., 2005; Iwamoto et al., 2013, 2014; Mata et al., 2015) via eye drops onto adult mice and rat eyes induced cell cycle reentry of Müller glial cells, which led to genesis of new mature retinal neurons. New cells first appeared in the inner nuclear layer and then migrated to the other layers (Webster et al., 2017, 2019). This observed inner nuclear migration is consistent with regenerative capacities in developing mice (Tackenberg et al., 2009; Webster et al., 2017). Cell cycle reentry was indicated by the presence of 5-bromo-2′-deoxyuridine (BrdU) positive cells observed after eye drop application of PNU-282987. The source of new BrdU positive cells was found to be Müller glia through lineage tracing with a transgenic TdTomato-Müller glia reporter line (Webster et al., 2017, 2019).

However, Müller glia do not express α7 nAChRs (Dmitrieva et al., 2007; Iwamoto et al., 2013, 2014; Webster et al., 2019). Of the cell types within the neural retina which contain α7 nAChRs, namely bipolar, some amacrine cells and retinal ganglion cells (Webster et al., 2017; Hall et al., 2019), acetylcholine (ACh) released from the starburst amacrine cells does not induce proliferation or interkinetic nuclear migration of Müller glia under physiological conditions. Instead, evidence was provided that PNU-282987 acts on the retinal pigment epithelium (RPE) when applied as eye drops (Webster et al., 2019). RPE cells contain α7 nACh receptors, are known to secret at least 20 different compounds under normal physiological conditions including growth factors and hormones, and are separated from ACh stimulation under normal physiological conditions by the outer limiting membrane (Grierson et al., 1994; Yoshii et al., 2007; Maneu et al., 2010). When PNU-282987 is administered as eyedrops into the eyes of adult mice and rats, it is hypothesized that PNU-282987 binds to alpha7 nAChRs on RPE directly outside the neural retina (Webster et al., 2019) to release signaling molecules onto the end feet of Müller glia, that then asymmetrically divide to produce Müller-derived progenitor cells (MDPCs). Evidence to support this hypothesis was provided by experiments in which RPE-J cell lines in culture were treated with 100 nM PNU-282987 and the resulting intraocular injection of supernatant was sufficient to produce cell cycle reentry of Müller glia in adult rodents *in vivo* (Webster et al., 2019).

This mechanism of generating new neurons from Müller glia is consistent with regeneration of the retina in adult zebrafish following injury. In Zebrafish, the *HB-EGF/Ascl1/Lin28a* pathway is found to be crucial in retinal regeneration following injury (Wan et al., 2012; Wan and Goldman, 2016). HB-EGF is one of many ligands which activate EGF receptors, others of which include EGF and TGFα (Wan et al., 2012). HB-EGF is localized to the point of damage in an injured zebrafish retina. It has been demonstrated that *HB-EGF* activates regeneration-associated gene *Ascl1*, which has been shown to modulate histone binding involved in dedifferentiation of neuronal cells (Vierbuchen et al., 2010). Ascl1 activates RNA binding protein Lin28a, which is known to inhibit microRNA *Let-7* (Yao et al., 2016). *Let-7* is involved in inhibition of proliferative abilities in adult Müller glia in zebrafish (Yao et al., 2016). When *Let-7* is inhibited, the *Wnt* pathway is active, leading to dedifferentiation of Müller glia to produce MDPCs (Yao et al., 2016). In this current study, evidence will be presented that these pathways are also involved in the PNU-282987 response in mammalian Müller glia.

This study was designed to explore the gene expression profiles involved in dedifferentiation of adult Müller glia cells to MDPCs in mammals. These studies demonstrate that exposure of cultured Müller glia cells to transwells of RPE cells treated with PNU-282987, will induce genesis of MDPCs. In addition, the results from this study support the hypothesis that the *HB-EGF/Ascl1/Lin28a* pathway is activated in Müller glia after stimulation of supernatant from PNU-282987 treated RPE adult rat cell lines. The results from transcriptomic analysis were compared to published pathways involved in retina development and/or retinal regeneration. Furthermore, the transcriptomics were used to describe potential interactions involved in the dedifferentiation of mammalian Müller glia. Additionally, it was demonstrated that communication between PNU-282987-activated RPE and Müller glia can induce progenitor-like fate in Müller glia culture, a process that does not typically occurs in adult mammals. Understanding these mechanisms is an important step in learning to control neurogenesis and regeneration in the adult mammalian retina.

## Materials and Methods

### Cell Culture

#### Culture of RPE Cell Lines

RPE cells derived from rats (RPE-J; 4-6 passages) were grown in Dulbecco’s modified Eagle’s medium containing 8% fetal bovine serum, 1% L-Glutamine, and 1% penicillin-streptomycin, using standard cell culture techniques and incubated in 33°C with 5% CO_2_. Propagation of RPE-J cell lines requires incubation lower than the standard 37°C. Cell culture reagents were obtained from Thermo Fisher Scientific (Waltham, MA, United States). RPE-J cell cultures were treated with dimethylsulfoxide (DMSO) (vehicle control) or PNU-282987 (100 nM; Sigma-Aldrich, St. Louis, MO, United States) for 24 h. Cells were plated in a T75 flask, 35 mm dish for immunocytochemistry or briefly in a 100 mm transwell dish (Corning, Inc., Corning, NY, United States) for co-culture with a cell line of Müller glia (rMC-1) and grown to 80% confluency. PNU-282987 or DMSO was applied to transwell or 35 mm dishes for 1 h. After thorough washing cells to remove any residual PNU-282987, cells were either placed in the co-culture system, or left for 72 h and “activated RPE supernatant” was collected. 100 nM PNU-282987 was used in this study, as previous dose-response studies from this lab have found 100 nM PNU-282987 to elicit the most robust proliferation response (Webster et al., 2017).

#### Culture of Müller Glia Cell Lines

Müller glia cells derived from rats (rMC-1; 4-6 passages) were grown in Dulbecco’s modified Eagle’s medium containing 8% fetal bovine serum, 1% L-Glutamine, and 1% penicillin-streptomycin, using standard cell culture techniques and incubated at 37°C with 5% CO_2_. After 3 days, cells were switched to 33°C with 5% CO_2_ so they could be co-cultured with RPE cells. The change in temperature had no effect on Müller glia growth and morphology if they were first cultured in 37°C with 5% CO_2_. Müller glia were co-cultured with RPE-J in transwells at 33°C with 5% CO_2_. Cell culture reagents were obtained from Thermo Fisher Scientific (Waltham, MA, United States). rMC-1 cell cultures remained untreated and were co-cultured with RPE-J cells that had previously been treated with DMSO (vehicle control) or PNU-282987 (100 nM; Sigma-Aldrich, St. Louis, MO, United States). rMC-1 cells were plated in a T75 flask, 35 mm dish for immunocytochemistry, or briefly in a 100 mm transwell dish (Corning, Inc., Corning, NY, United States) and grown to 80% confluency for coculture with RPE-J. For RNA extraction, Müller glia were exposed to treated RPE via transwells for set times (8, 12, 24, or 48 h). When these co-culture exposure times were completed, cells were collected using 1:250 trypsin-EDTA and gently pelleted using centrifugation. For immunocytochemistry, Müller glia were grown to 30% confluency and treated with activated RPE-J supernatant after treatment with DMSO or PNU-282987 for 0, 8, or 48 h or were incubated in transwells containing treated RPE-J cells for the same amount of time.

### RNA Extraction and RNAseq Analysis

Direct-zol RNA Miniprep kit (R2051; Zymo Research) was used for RNA extraction following the protocol provided. RNA from Müller glia cells in culture was collected in RNase-free water and stored at −80°C or on dry ice for overnight shipment to GeneWiz. GeneWiz performed RNA-seq next generation sequencing (NGS) using the Illumina NextSeq 550 high-output platform (Illumina, San Diego, CA, United States). Sequence reads were trimmed using Trimmomatic v.0.36. Trimmed sequence reads were mapped to the *Rattus norvegicus* Rnor6.0 reference genome available on ENSEMBL. Unique gene hit counts were calculated using featureCounts (subread package v.1.5.2). GeneWiz provided Deseq2 bioinformatics analysis. Pathway analysis was additionally performed in this lab using Reactome (Fabregat et al., 2014).

### Quantitative Reverse Transcription Polymerase Chain Reaction (qRT-PCR)

qRT-PCR was preformed using the SuperScript III Platinum One-Step kit with ROX (11745500, Thermo Fisher) with RNA collected from each of the five timepoints, as described previously. 50 ng of RNA was used for each reaction with a RIN ≥ 7 and set up in 10 μL reaction volumes, as per the manufacturers protocol. IDT and Thermo pre-designed primer/probe assays were used (Table 1) and efficiencies were validated for each primer/probe set to be between 90 and 110% (Supplementary Table S1). Each sample was run in triplicate and each assay was repeated in triplicate. For relative comparison of gene expression, the real-time results were analyzed using the comparative Ct method (2^–ΔΔ*Ct*^) normalized to a *Gapdh* housekeeping control. Negative controls were performed for each RNA sample and each primer/probe set in which no Reverse Transcriptase and no RNA template were added, respectively.

**TABLE 1:** Primer/probe assays used.

Gene symbol	Gene assay ID
HB-EGF	Rn.PT.58.9646801
Ascl1	Rn.PT.58.17863661.g
Lin28a	Rn.PT.58.33962866
Fgf11	Rn.PT.58.5164978
Fgf9	Rn.PT.58.10285426
Sox9	Rn.PT.58.29440750
Fzd9	Rn00596271_s1
Mmp9	Rn00579162_m1
Gli3	Rn01538495_m1
Bcat1	Rn.PT.58.44552624
Bmp4	Rn.PT.58.36829962
Gapdh	Rn.PT.39a.11180736.g

*Summary of primer probes used for qRT-PCR Taqman Assay.*

### Immunofluorescence for Müller Glia in Culture

Müller glia cells were grown to 30% confluency on 35 mm dishes (Thermo Fisher Scientific, Waltham, MA, United States) and treated for 0, 8, and 48 h with activated RPE supernatant or in transwells containing PNU-282987 treated RPE cells. After treatment, Müller glia cells were fixed using 4% paraformaldehyde (PFA) for 10 min at room temperature. PFA was then removed, and cells were washed 3x with 1X Phosphate Buffer Saline (PBS). To increase permeabilization, 0.5% Triton X-100 in 1X Tris Buffer Saline (TBSTr) was added and cells were incubated at room temperature for 15 min. TBSTr was removed and replaced with TBSTr with 10% Normal Goat Serum (NGS) for 4 h to minimize non-specific binding. Primary antibodies were diluted at a ratio of 1:300 in 1X TBS + 0.05% Triton X-100 with 1% NGS and incubated overnight at 4°C. Primary antibodies (Table 2) were then removed and cells were washed 3x with 1X TBS + 0.05% Triton X-100 for 10 min. Fluorescent dye labeled secondary antibodies (Table 2) were added for 2 h at room temperature in the dark, followed by Hoescht counterstain (1:10,000) for 10 min at room temperature. Cells were again washed 3x with 1X TBS. Fluorescently labeled cells were viewed using Nikon C2 + scanning confocal microscopy. Antibodies against OTX2, VSX2, and Nestin were used to identify the presence of retinal progenitor cells, and Vimentin was used to label Müller glial cytoskeleton as a positive control. All reagents were obtained from Thermo Fisher Scientific (Waltham, MA, United States) and antibodies were obtained from Abcam^®^, Inc. (Cambridge, MA, United States). In control studies, experiments were conducted to determine the specificity of all antibodies used. Antibodies used in this study originated from rabbit, mouse, goat, donkey, or sheep. Antibody processing was performed on untreated Müller glia rMC-1 cells as well as in experimentally treated cells of the same type to determine if positive labeling occurred in untreated controls. The number of immunostained cells in individual culture wells were normalized to the total number of cultured cells in each culture well that were counterstained with Hoescht. N’s of 5-10 were collected for each antibody used in this study and counts were averaged. In control studies, cells were processed with the primary antibody omitted. No significant epifluorescence was observed if the primary antibody was omitted.

**TABLE 2:** Antibodies used in immunocytochemistry of rat-derived cell lines.

1° Antibody	Immunogen	Cat. number	1° Dilution	2° Antibody	Cat. number	2° Dilution
Mouse α-Vimentin	Müller glia cytoskeleton	ab8978	1:300	Goat α-mouse	ab150120	1:300
Mouse α-Nestin	Retinal progenitor	ab6142	1:300	Goat α-mouse	ab150120	1:300
Rabbit α-OTX1/2	Retinal progenitor	ab92515	1:300	Goat α-rabbit	ab150088	1:300
Sheep α-CHX10 (VSX2)	Retinal progenitor	ab16141	1:300	Donkey α-sheep	ab150177	1:300

*Summary of primary and secondary antibodies used to test the presence of retinal progenitor cell markers in Müller glia which were exposed to supernatant of RPE cells treated with DMSO vs. PNU-282987.*

### Statistical Analysis

Single comparisons involved a Student’s paired *t*-test for all culture experiments. For experiments requiring multiple comparisons, one-way ANOVA with *post hoc* analysis using the Holm-Bonferroni correction was performed. A Wald test was performed to generated *P*-values and log_2_ fold changes of RNA-seq results. Results were considered significant if *P* ≤ 0.05 and log_2_ fold changes were > 1 or < −1. Statistical analysis for RNA-seq was provided by GeneWiz. Heat map samples of significant genes were determined by systematic sampling using the equiprobability method to in order to accurately represent total sDEGs at each timepoint.

### Bioinformatics

Bioinformatics performed by GeneWiz provided significant differentially expressed genes (sDEGs) and Volcano plots using the Wald test to determine DEGs with a *P*-value ≤ 0.05 and log_2_ fold changes > 1 or < −1. Heat maps were generated using Morpheus and Violin Plots using Plotly. Reactome was used to determine biological processes associated with pathways of identified genetic markers. Binary Alignment Sequences (BAMs) were generated by GeneWiz using STAR Aligner v.2.5.2b. All other tables and graphs were generated using Excel.

## Results

### Experimental Design

**FIGURE 1:**
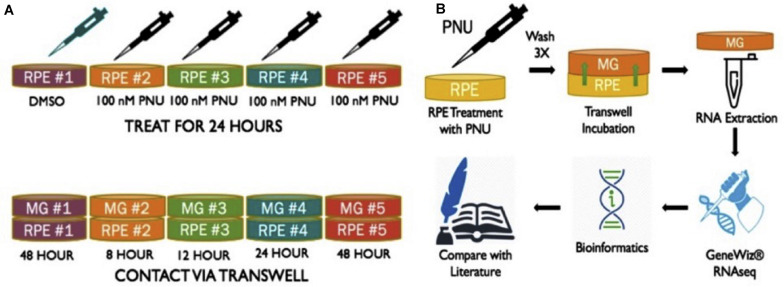
Schematic of methods performed to obtain RNA from Müller glia exposed to PNU-282987 treated RPE cells. **(A)** (1) RPE-J treated for 24 h with DMSO as a vehicle control. (2-5) RPE-J cells treated for 24 h with PNU-282987. PNU-282987 was removed and thoroughly washed away with PBS before being replace with normal media. **(B)** After PNU-282987 treatment and thorough rinsing, RPE were exposed to rMC-1 cells via transwells and allowed to incubate for 8, 12, 24, and 48 h. RNA was extracted from Müller glia and sent to GeneWiz for RNA sequencing. Some bioinformatics was performed to determine log_2_ fold changes of identified RNA sequences compared to DMSO controls.

Previous studies from this lab found that supernatant collected from RPE cells treated with PNU-282987 (activated RPE) was sufficient to produce cell cycle reentry of the adult mammalian Müller glia in the retina when the supernatant was injected into the vitreal chamber of adult rodents *in vivo*. These same studies demonstrated that BrdU positive Müller glia dedifferentiate into MDPCs (Webster et al., 2017, 2019). To identify changes in Müller glia gene expression after exposure to activated RPE supernatant, RNA-seq was performed (GeneWiz Inc., NJ, United States) from total RNA extracted from Müller glia. rMC-1 Müller glial cell lines (Kerafast ENW001) were exposed to activated RPE via 75 mm Transwell (Corning 3419) dishes (Figure 1). RPE cells were cultured for 8, 12, 24, and 48 h in normal media (8% FBS DMEM with L-glutamine and Penicillin/Streptomyocin, Gibco 11971025) after exposure to 100 nM PNU-282987 for 24 h, followed by thorough rinsing with PBS. Previously, RPE culture was treated with PNU for 24 h, washed, and subsequently left in normal media for 72 h before injection in the mouse eye. This timeline induced robust BrdU incorporation (Webster et al., 2019). Subsequent experiments analyzed 12, 24, and 48 h cultures, which all showed some level of BrdU incorporation in the mouse retina (unpublished). This information was used to select the appropriate time points in order to see early changes in Müller glia transcript as well as changes leading up to the maximum response attainable while maintaining healthy cell cultures. In some 75 mm transwell dishes, RPE-J cells were treated with 1% DMSO for 24 h instead of 100 nM PNU-282987 to act as a vehicle control. Figure 1 summarizes the procedures used to perform these experiments.

### RNA Sequencing of Müller Glia Exposed to PNU-282987-Treated RPE Supernatant

**FIGURE 2:**
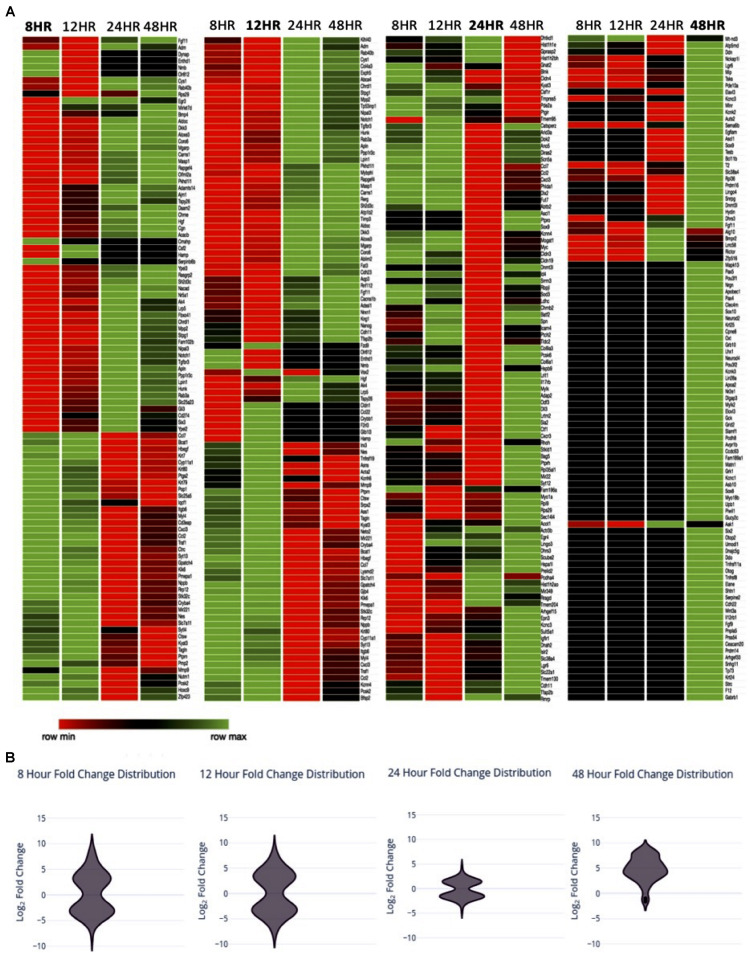
Expression changes over time **(A)** Heat map with of sDEGs from Müller glia RNA expression changes 8, 12, 24, and 48 h post-exposure to PNU-282987 treated RPE cells, compared to 48-h DMSO control conditions. sDEGs represented include a linear sample of total sDEGs per timepoint. Created using Morpheus. **(B)** Violin distribution plots of sDEGs per timepoint at the timepoint represented.

In order to investigate transcriptional changes in Müller glia following the treatment with PNU-282987 described in Figure 1, RNA-seq analysis of a Müller glial cell line was performed. Each sample was sequenced by GeneWiz using the Illumina NextSeq 550 high-output platform (Illumina, San Diego, CA, United States). Log_2_ fold expression changes were obtained in approximately 15,000 identified genes total for the four timepoints. 1050 significantly differentially expressed genes (sDEGs) were identified, and were compared to literature identifying genes of interest known to be involved in dedifferentiation of Müller glia in teleost fish upon retinal injury (Hitchcock et al., 1996; Fischer and Reh, 2001; Ramachandran and Zhao, 2011; Meyers et al., 2012; Wan et al., 2012; Centanin and Wittbrodt, 2014; Goldman, 2014; Gorsuch and Hyde, 2014; Yao et al., 2016), as well as those involved in early development of the retina in vertebrate mammals such as mouse and rat (Bhattacharjee and Sanyal, 1975; Wang et al., 2001; Dhomen et al., 2006; Locker et al., 2006; Nelson et al., 2009; Phillips et al., 2011; Wright et al., 2015; Xia and Ahmad, 2016). Teleost fish, specifically zebrafish, were chosen as a comparative focal point in this study based on the zebrafish’s ability to regenerate the retina in adulthood following injury, the extensive availability of research published on this topic, and the finding that PNU-282987 induced neurogenesis in rats and mice are generated from Muller glia (Webster et al., 2017, 2019). For comparison, 100 molecules of interest per timepoint were chosen as a focus for this study, illustrated in the heat map in Figure 2A. The 100 genes per timepoint chosen to represent the sDEG data were identified by systematic sampling using the equiprobability method to accurately represent the distribution of log_2_ fold changes identified as significant at the timepoint.

In Table 3, the number of sDEG at each collection time point is summarized as well as the direction of expression for the genes at each collection time point and any change in gene expression at later time points. For example, at the 8-h time point, 409 DEGs were found to be statistically significant (*P*-value < 0.05, log_2_ fold change > 1 or < −1) compared to DMSO control. At 8 h, 240 of the 409 DEGs were down-regulated compared to control (log_2_ fold change between −1.81 and −8.01), while 169 (41.3%) were up-regulated (log_2_ fold change between 1.78 and 8.56). The majority of sDEGs that were down-regulated at 8 h reversed and were up-regulated by 24 and 48 h post-treatment. Conversely, the majority of sDEGs that were up-regulated at 8 h were down-regulated by 24 and 48 h post-treatment. As seen in Table 3, the reversal of gene expression at later time points occurred for most genes, indicating these genes are expressed for a relatively narrow window of time and are then actively reversed. This “on/off” type of behavior for the genes is a typical pattern for genes involved in growth, development and dedifferentiation.

**TABLE 3:** Changes of significant differential gene expression (sDEG) that occurred when Muller glia cells were cultured in transwells with PNU-282987 treated RPE cells for various amount of time.

First appearance of DEG	Number of sDEGs	Up/down expression	Change of expression
8 h	404	240/409↓	Reverses expression at 24
		169/409↑	and 48 h.
12 h	420	237/420↓ 183/420↑	Reverses expression at 24 and 48 h.
24 h	272	155/272↓ 117/272↑	Reverses expression at 48 h or no change occurs.
48 h	339	38/339↓ 301/339↑	N/A

As seen in Table 3, most of the sDEGs first expressed 48 h post-treatment compared to the DMSO control were up-regulated and had much larger fold changes than genes expressed at other time points (log_2_ fold change between 1.0 and 8.71). These genes were specifically analyzed for their potential contribution toward dedifferentiation of Muller glia into MDPCs.

### Pathway Analysis of Differentially Expressed Genes

**FIGURE 3:**
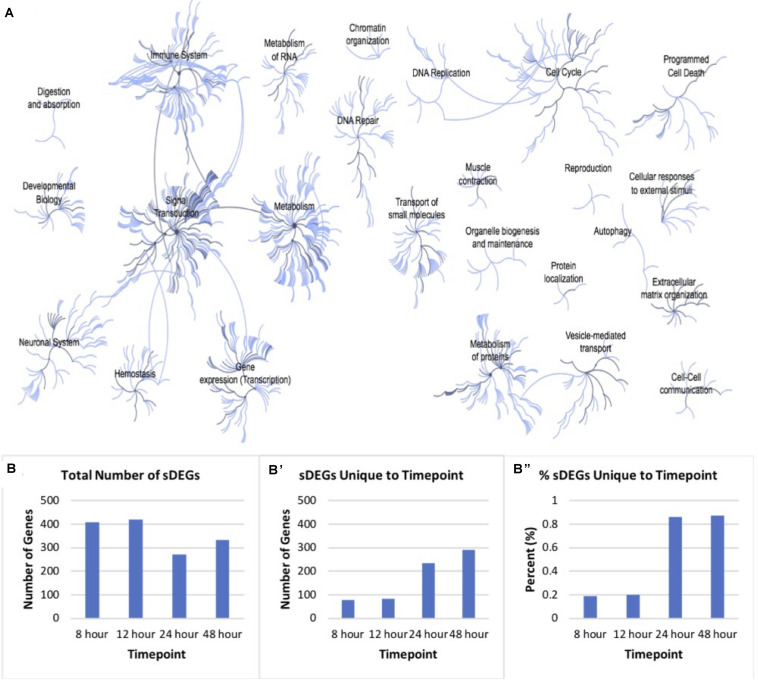
Pathway analysis of differentially expressed genes. **(A)** Reactome map display of the distribution of biological processes in which significant differentially expressed genes identified via mRNA sequencing of timepoints listed in Figure 2 are involved in rat. **(B)** Distribution of total significantly expressed genes 8, 12, 24, and 48-h post-exposure to PNU-282987 treated RPE supernatant compared to DMSO control. **(B′)** sDEGs which were only found to be significant at the corresponding timepoint. **(B′′)** Percent of sDEGs which were unique to a timepoint compared to the total number at that timepoint.

In total, there was a total of 1050 unique sDEGs when all timepoints were considered. This list was analyzed using Reactome pathway analysis in order to understand which biological processes in *Rattus norvegicus* these transcripts are known to be involved in Figure 3A. 120 genes involved in signal transduction were identified. Specifically, genes involved in signaling by TGF-β, Notch, Wnt, MAPK, Hippo, and Hedgehog pathways were identified in the sDEGs analyzed. These pathways are all known to be involved in retinal regeneration in vertebrates like zebrafish and chick (Hayes et al., 2007; Tackenberg et al., 2009; Meyers et al., 2012; Wan and Goldman, 2016; Yao et al., 2016). As well as signal transduction, genes involved in developmental pathways, metabolism and immunology were identified. The majority of genes involved in development pertain to pathways in nervous system development and nervous system function. Genes involved in early development were particularly relevant for this study as fully differentiated Müller glia do not typically express early neuronal development genes. 14 genes involved in mitosis were identified, as well as genes involved in G1/S DNA damage checkpoints. Though the analysis was performed on RNA extracted from mitotically active cell lines, normalization of values compared to the control condition accounted for the genes involved in immortalization.

Although genes involved in development and differentiation was the focus of this study, genes involved in metabolism were also identified that are known to be involved in metabolic processes such as protein synthesis, regulation of insulin-like growth factor (IGF) transport, RNA processing, integration of energy metabolism, chromatin modification, gene silencing, and protein localization. In addition, a large immune response was also detected through Reactome pathway analysis, including significant expression changes of inflammatory genes like macrophage expressed 1 (*mpeg1*) and cytokine expressed *Csf1R* (Huynh et al., 2013; Mitchell et al., 2018). In zebrafish, acute inflammation occurs in the damaged tissue and is known to promote dedifferentiation and proliferation in response to injury in the retina (Silva et al., 2020). However, the change of gene expression pertaining to the immune response was not the focus of this study.

Figure 3B shows the number of sDEGs at each timepoint (B), as well as the number (B′) and percent (B′′) of those genes which were unique at each timepoint. For example, the 8 h timepoint shared 80.7% sDEGs of all with the 12 h timepoint, and only 2.2% with the 24 h timepoint and 3.9% with the 48 h timepoint. Overall, the 24 and 48 h timepoints had a more unique transcript than the other conditions. *In vivo*, these two timepoints produced the maximal number of BrdU + cells following PNU-282987 treatment and was the time when retinal progenitor markers began to be expressed (Webster et al., 2017, 2019). The patterns of gene expression changes in this data are consistent with what we would expect in cells that are proliferating (early) and then dedifferentiating (late).

To understand how the up- and down-regulation of these genes may contribute to dedifferentiation of the Müller glia, known pathways of retinal regeneration in zebrafish and chick, as well as those known to be involved in development of the mammalian retina were compared to the results obtained from RNA-seq results (Table 4). Table 4A shows sDEGs at the 8 h timepoint that are involved in genes associated with differentiation and proliferation. Up-regulation of β-catenin, a co-transcriptional regulator in the *Wnt/*β*-Catenin* pathway of proliferation (Gallina et al., 2016), HB-EGF, a ligand known to initiate retinal regeneration in zebrafish (Wan et al., 2012), and *Mmp9*, an initiator of *Wnt* signaling through the Hedgehog pathway of retinal regeneration in chick (Kaur et al., 2018) was observed. Down-regulation of *Bmp4*, a transcription factor associated with adult Müller cell identity as well as down-regulation of *Wnt* inhibitors such as transcription factor *Dkk3*, MicroRNA *Let7d* and hedgehog transcription factor *Gli3* were observed at 8 h as well (Nakamura et al., 2007; Kaur et al., 2018).

**TABLE 4:** Molecules of interest in Müller glia dedifferentiation.

Molecule	Description	Gene ID	RNA-Seq Log2 fold change
**(A) Top DEGs following 8 h PNU-282987 treated RPE supernatant exposure**			
Bcat1	β-Catenin	ENSRNOG00000015514	2.84805029
Bmp4	Bone morphogenic protein 4	ENSRNOG00000009694	–1.43867
Fgf11	Fibroblast growth factor 11	ENSRNOG00000014882	–2.0627042
Gli3	GLI family zinc finger 3	ENSRNOG00000014395	–1.1229905
Hbegf	Heparin binding epidermal like growth factor	ENSRNOG00000018646	1.914186199
Hoxc9	Homeobox C9	ENSRNOG00000016199	1.108708692
Hunk	Hormonally up-regulated Neu-associated kinase	ENSRNOG00000002092	–6.1308569
Tgfbr3	Transforming growth factor beta receptor 3	ENSRNOG0000002093	–2.1373061
Lrp5	LDL receptor related protein 5	ENSRNOG00000015911	–1.9236029
Dkk3	Dickkopf WNT signaling pathway inhibitor 3	ENSRNOG00000016343	–2.1457008
Mmp9	Matrix metallopeptidase 9	ENSRNOG00000017539	2.56663589
Mirlet7d	MicroRNA Let-7d	ENSRNOG00000035594	–3.01539
Notch1	Notch 1	ENSRNOG00000019322	–2.0502037
Ptprn	Protein tyrosine phosphatase receptor type N	ENSRNOG00000019587	1.90681708
**(B) Top DEGs following 12 h PNU-282987 treated RPE supernatant exposure**			
Bcat1	β-Catenin	ENSRNOG00000015514	3.14109256
Fgf11	Fibroblast growth factor 11	ENSRNOG00000014882	–3.108506158
Fzd9	Frizzled class receptor 9	ENSRNOG00000015365	1.845663361
Hbegf	Heparin binding epidermal like growth factor	ENSRNOG00000018646	1.91776018
Hunk	Hormonally up-regulated Neu-associated kinase	ENSRNOG00000002092	–5.1766766
Tgfbr3	Transforming growth factor beta receptor 3	ENSRNOG00000002093	2.0120589
Nanog	Homeobox transcription factor Nanog	ENSRNOG00000008368	–7.2407881
Dkk3	Dickkopf WNT signaling pathway inhibitor 3	ENSRNOG00000016343	–2.0962496
Mmp9	Matrix metallopeptidase 9	ENSRNOG00000017539	4.19752803
Notch1	Notch receptor 1	ENSRNOG00000019322	–1.9327218
Ptprn	Protein tyrosine phosphatase receptor type N	ENSRNOG00000019587	2.04237659
Nestin	Nestin	ENSRNOG00000018681	1.66442802
Vsx2	Visual system homeobox 2	ENSRNOG00000011918	1.31959804
**(C) Top DEGs following 24 h PNU-282987 treated RPE supernatant exposure**			
Ascl1	Ashaete-Scute homolog 1	ENSRNOG00000004294	1.948558807
Cxcl3	C-X-C motif chemokine ligand 3	ENSRNOG00000028043	–1.143854
Ano5	Anoctamin 5	ENSRNOG00000015972	–4.5141164
Myc	MYC proto-onco gene, BHLH transcription factor	ENSRNOG00000004500	–1.1139704
Sox9	SRY-box 9	ENSRNOG00000002607	1.38067151
Rps29	Ribosomal protein S9	ENSRNOG00000032542	2.38718436
Ptpro	Protein tyrosine phosphatase receptor type O	ENSRNOG00000006231	2.87816537
Map2k6	Mitogen-activating protein kinase, kinase 6	ENSRNOG00000004437	2.40172969
Cntd1	Cyclin N-terminal domain containing 1	ENSRNOG00000051150	2.40172969
**(D) Top DEGs following 48 h PNU-282987 treated RPE supernatant exposure**			
Ascl1	Ashaete-Scute homolog 1	ENSRNOG00000004294	3.998554703
Pou3f2	POU class 3 homeobox 2	ENSRNOG00000006908	4.42232
Myt1	Myelin transcription factor 1-like protein	EN5RNOG00000017346	4.355205
Elavl3	ELAV like RNA binding protein 3	ENSRNOG00000013752	2.341450786
Fgf11	Fibroblast growth factor 11	ENSRNOG00000014882	1.563336091
Fgf9	Fibroblast growth factor 9	ENSRNOG00000011471	7.160392996
Lin28a	Lin-28 homolog A	ENSRNOG00000060320	4.770246202
Sox9	SRY-box 9	ENSRNOG00000002607	4.527716699
Wnt3a	Wingless-type MMTV integration site family, Member 3A	ENSRNOG00000003039	7.53479911
Sox10	SRY-related HMG-box Gene 10	ENSRNOG00000011305	3.98085796
Sox8	SRY-related HMG-box Gene 8	ENSRNOG00000018841	5.8577355

***(A–D)** Lists log_2_ fold changes of RNA extracted from treated Müller glia.*

Table 4B shows sDEGs that occurred at 12 h. The pattern of expression at 12 h was very similar to the 8 h timepoint, with up-regulation of β*-catenin*, *HB-EGF*, and *Mmp9*, as well as down-regulation of *Dkk3*. In addition, *Fzd9*, a frizzled receptor which *Wnt* ligands bind (Dijksterhuis et al., 2014), was up-regulated at 12 h, which could point to an increase in *Wnt* signaling at this time. In addition, early retinal progenitor markers *Vsx2* and *Nestin* (Wright et al., 2010; Lee et al., 2012), were also upregulated at 12 h.

Table 4C shows sDEGs that occurred at 24 h and illustrates expression of genes for the first time. Specifically *Ascl1* and the co-transcriptional regulator Lin28a, which are involved downstream of *HB-EGF*, activates *Wnt* signaling in zebrafish regeneration (Wan et al., 2012). In addition, the retinal progenitor marker *Sox9* is expressed for the first time after 24 h of treatment (Poché et al., 2008).

Table 4D shows sDEGs that occurred at 48 h. There is up-regulation of *Ascl1* again as well as *Lin28a*, *Ascl1* recruiters *Pou3f2* and *Myt1* (Chanda et al., 2014), and the retinal progenitor marker *Sox9*. However, new up-regulation occurs as well, such as fibroblast growth factors *Fgf9* and *Fgf11*. Fgf genes are involved in the regeneration response in zebrafish and chick retina (Fischer and Reh, 2001; Wan and Goldman, 2017) and have been shown to be regulators of *Wnt* signaling (Tang et al., 2019). These data highlight the conservation of gene expression profiles commonly observed in other models of retinal regeneration to the PNU-282987 activated mammalian system and demonstrate when expression occurs. Understanding the timing of these gene expression allowed us to hypothesis what signaling pathways are involved in Müller glia cell proliferation after exposure to PNU-282987 treated RPE cells.

**FIGURE 4:**
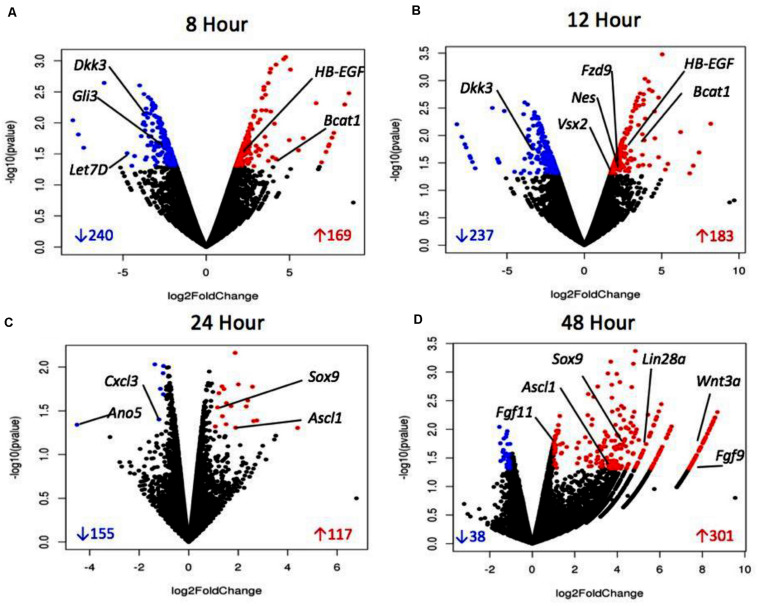
Volcano plots of sDEGs at 8 h vs. control **(A),** 12 h vs. control **(B)**, 24 h vs. control, **(C)** and 48 h vs. control **(D)**. Plots demonstrate all DEGs as dots based on their fold change, with down-regulated sDEGS in blue and up-regulated sDEGs in red.

Volcano plots (Figure 4) represent a distribution of the log_2_ fold changes seen at each timepoint. The black points represent differentially expressed genes, whereas blue points are those which were significantly up-regulated and red were significantly down-regulated. The volcano plots for 8 and 12 h show a consistent distribution of both significantly up- and down-regulated genes. The plot for 24 h shows fewer significant genes overall, with many genes showing a similarity in expression. The 48 h plot shows a striking disparity between up- and down-regulated differentially expressed genes, with the majority being up-regulated and to a higher degree than what is seen at other timepoints. This data overall points to up-regulation of genes early on which are related to proliferation and dedifferentiation of Müller glia and down-regulation of genes known to prevent proliferation. In later timepoints, up-regulation of genes associated with asymmetrical division of Müller glia that produce cells with a retinal progenitor-like fate are seen.

### qRT-PCR Confirmation of Differentially Expressed Genes

**FIGURE 5:**
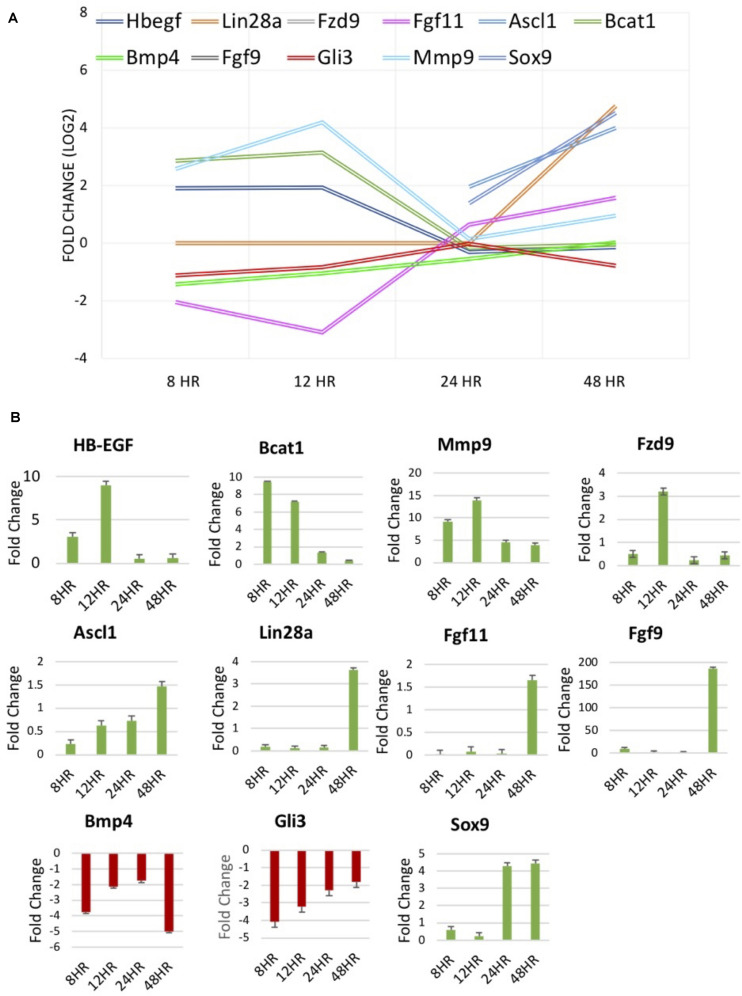
**(A)** RNA-seq results of molecules of interest identified in literature to be involved in regeneration of the zebrafish and chick retina and mammalian retinal development. **(B)** Fold change [2^– –^ΔΔCT] results of qPCR verification of molecules of interest in **(A)**.

qRT-PCR was performed to validate RNA sequencing results for several genes of interest thought to be involved in dedifferentiation pathways. RNA-seq data and qRT-PCR data were displayed for factors at differing treatment timepoints (Figure 5). *BMP4*, a protein coding gene in the TGFβ family, known to be down-regulated in chick MDPCs (Todd et al., 2017), is found to be down-regulated at 8 h in RNA seq results as well as all other timepoints in qRT-PCR. *HB-EGF*, found to be up-regulated at 8 and 12 h in RNA seq results, was confirmed to follow the same trend via qRT-PCR, as well as qRT-PCR confirmation of *Ascl1* and *Lin28a* up-regulation at 48 and 24/48 h, respectively. These finding are consistent with the pathway of retinal regeneration in zebrafish in which *HB-EGF* activation at the site of injury leads to the activation of *Ascl1* to then recruit *Lin28a* (Ramachandran and Zhao, 2011; Wan et al., 2012; Wan and Goldman, 2016). *Bcat1* and *Fzd9*, up-regulated at 8 and 12 h, and *Fgf9/Fgf11*, up-regulated at 48 h in RNA seq, followed the same trend in qRT-PCR verification. *Fzd9* is a receptor which binds *Wnt* ligands (Meyers et al., 2012; Wan et al., 2012; Tappeiner et al., 2016). *FGF9* and *FGF11* up-regulated in Müller-derived progenitor cells in other vertebrates (Fischer and Reh, 2001; Hochmann et al., 2012; Wan and Goldman, 2016). *Mmp9* and *Gli3*, components of the sonic hedgehog (Shh) pathway that are up- and down-regulated respectively early in zebrafish regeneration (Wan and Goldman, 2016; Kaur et al., 2018) were up- and down-regulated at 8 and 12 h in RNA-seq, confirmed in qRT-PCR. Finally, retinal progenitor marker *Sox9* (Young, 1985; Webster et al., 2017) was up-regulated at 24 and 24 h, which is confirmed in qRT-PCR results. This data provides evidence to support findings in the RNA-seq results.

### Dedifferentiation of Müller Glia to Müller-Derived Progenitor Cells

**FIGURE 6:**
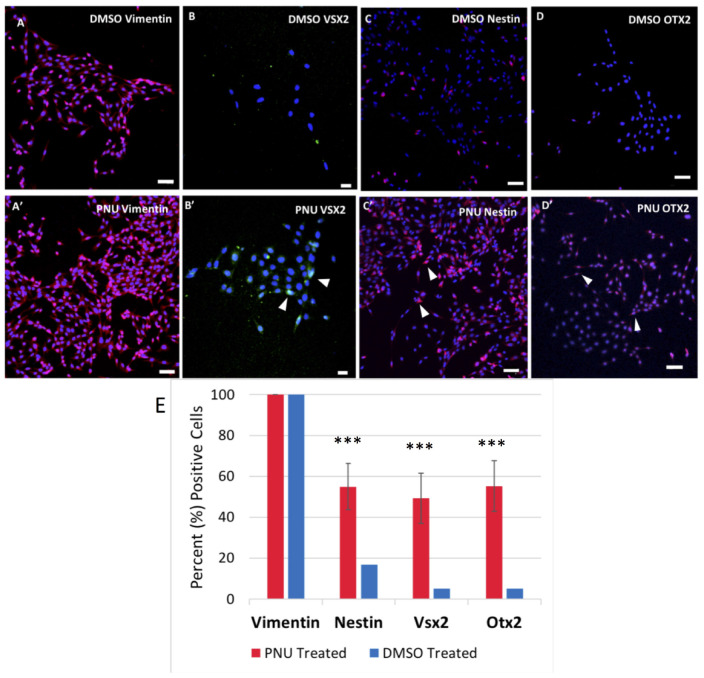
Immunocytochemistry of Müller glia after 48-h exposure to PNU-282987 treated RPE supernatant. Scale bars represent 100 microns. **(A,A′)** Müller glial cytoskeletal component, Vimentin, used as a control in DMSO and PNU-282987 treated rMC-1. **(B,B′)** Retinal progenitor marker VSX2 is identified in treated Müller glia. Arrows indicate VSX2 + single nuclei. **(C,C′)** Retinal progenitor marker Nestin labels cytoskeleton of treated Müller glia. Arrows indicate single Nestin + cells. **(D,D′)** Transcription factor OTX2 labels both nucleus and cytoskeleton of treated Müller glia. Arrows indicate OTX2 + cells. Scale bar represents 100 microns. **(E)** Quantification of cells positive for progenitor markers. A one-way ANOVA was performed to determine statistical significance. All three progenitor cell markers were found to be statistically significant when compared to DMSO controls (***P-value < 0.001) in PNU-282987 treated cells.

To provide evidence that MDPCs were produced as a result of signaling molecules released from the RPE, IHC was performed. To identify progenitor cell markers in Müller glia, rMC-1 cultures were exposed to activated RPE supernatant for 48 h in some studies, or allowed to incubate in transwells containing RPE cells that had previously been treated with PNU-282987. Both methods produced statistically similar changes in Müller glia. Following incubation with the supernatant or when with treated RPE cells in transwells, cells were labeled with antibodies against vimentin, VSX2, nestin and OTX2. Figure 6 illustrates confocal images obtained when the supernatant from PNU-282987 treated RPE cells were added to culture dishes of Müller glia, as the resolution and clarity of images obtained in culture dishes was higher than images of Müller glia attached to the mesh on transwells (Figure 6). Vimentin, a Müller glia marker found in the unaltered adult retina (Tackenberg et al., 2009), was used as a control and labeled all cells in both the treated or untreated conditions (Figures 6A,A′). Nestin, expressed in neural precursor cells in the developing retina (Božanić et al., 2006), labeled Müller glia when introduced to the supernatant from PNU282987 treated RPE cells to a significantly greater degree than that found in control cells (Figures 6B,B′). VSX2, found in prenatal eye cultures in neurospheres with neurogenic potential (Wright et al., 2010), immunostained significantly more Müller glia than in control cells (Figures 6C,C′). Finally, OTX2, another retinal progenitor marker widely accepted to represent stem-like qualities in the developing retina (Schmitt et al., 2009), immunostained significantly more treated Müller glia than in untreated cells (Figures 6D,D′). Müller cells with positive staining for retinal progenitor markers provide evidence that Müller glia dedifferentiate to a progenitor-like fate when exposed to signaling molecules released from PNU-282987 treated RPE cells. This result indicates that PNU-282987 induces a response in Müller glia that is not typically seen in adult mammalian neurons.

### Summary of sDEGs Involved in Dedifferentiation of Müller Glia to MDPCs

**FIGURE 7:**
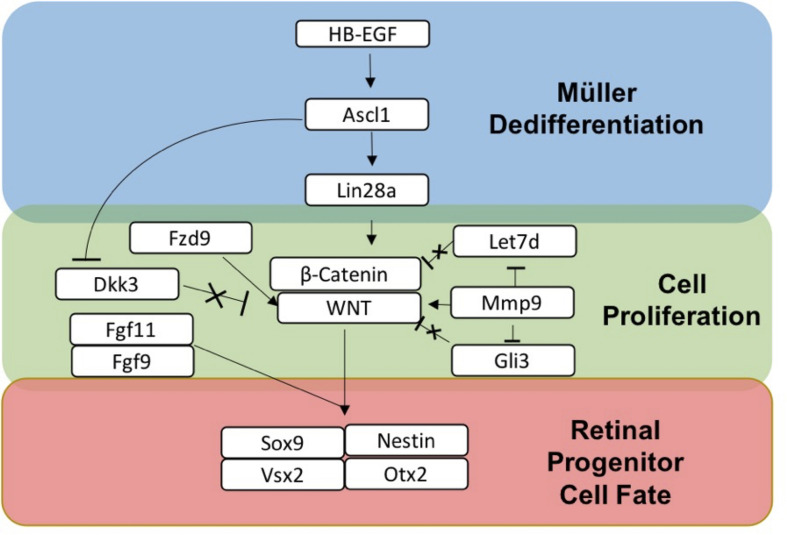
Summary of gene expression associated with dedifferentiation of Müller glia to Müller-derived progenitor cells in adult mammals. *HB-EGF* is up-regulated when Müller glia are exposed to RPE supernatant treated with PNU-282987.

Based on the transcriptomics and IHC marker data, a summary of sDEGS involved in the dedifferentiation of Müller glia to retinal progenitor cells following activated RPE supernatant treatment is outlined in Figure 7. This figure was constructed using the expression patterns as well as the timing of expression. For example, *HB-EGF*, an EGFR ligand (8, 12 h up-regulation), is known to work upstream of *Ascl1* (24, 48 h up-regulation), a basic helix-loop-helix transcription factor, which then activates RNA binding protein *Lin28a* (48 h up-regulation) to allow for dedifferentiation in the zebrafish retina (Wan and Goldman, 2016). MicroRNA *Let-7* (Down-regulation at 8 and 12 h) is shown to inhibit dedifferentiation in the Müller glia in the adult zebrafish prior to injury (Wan et al., 2012; Kaur et al., 2018). *Mmp9* (8, 12-h up-regulation) regulates *Ascl1* and is found early in dedifferentiation (Locker et al., 2006). Also, *Mmp9* along with *Gli3* are Shh pathway regulators of Let-7 in Zebrafish regeneration (Wan and Goldman, 2016; Kaur et al., 2018) *Fzd9* (12 h up-regulation) is a *Wnt* receptor known to be activated in *HB-EGF/Ascl1* signaling (Ramachandran and Zhao, 2011). Finally, *B cat1* signaling (Up-regulated at 8 and 12 h, later in qRT-PCR data) is both required in dedifferentiation to and maintenance of retinal progenitors produced in zebrafish (Meyers et al., 2012). β*-Catenin* also binds to *Lin28a* promotor to activate transcription (Yao et al., 2016). *Wnt/*β*-catenin* signaling is the pathway that drives the production of Müller-derived progenitor cells in chick (Gallina et al., 2016) as well as vertebrate eye development (Fujimura, 2016). The retinal progenitor marker, *Sox9* (up-regulated at 24 and 48 h) as well as progenitor markers Nestin, OTX2 and VSX2, identified in IHC, provide evidence of a MDPC-like fate.

## Discussion

In this study, we show that activated RPE supernatant initiates transcriptional changes in Müller glia that lead to gene expression profiles resembling that of dedifferentiated Müller glia and MDPCs including activation of genes involved the *HB-EGF/Ascl1/Lin28a* pathway. Activation of these genes were induced through signaling molecules released from PNU-282987 treated RPE cells, which has not been demonstrated before. Heat maps were generated comparing sDEG fold changes at experimental timepoints vs. control. Comparison of significant fold changes at 8/12 h reveals a distinct pattern of on/off behavior by sDEGs which were turned on at 8/12 h at off at 24/48, and vice versa. A vast change occurs at 48 h in which the majority of sDEGs are turned on with higher fold changes than seen at any other timepoint. Most sDEGs are unique to this timepoint. This pattern is similar to the change in gene expression pattern observed in the regenerating zebrafish and chick retina following injury. In zebrafish, proliferation inhibitors are turned off and signal transduction genes are turned on temporarily to allow for proliferation of Müller glia. Expression changes to upregulation of retinal progenitor markers later on in newly derived MDPCs (Fischer and Reh, 2001; Wan and Goldman, 2016; Todd et al., 2017).

Reactome analysis revealed significant changes in gene expression of sDEGs throughout the study in signal transduction pathways including *Wnt*/β-Catenin, TGF-β, Notch, MAPK, Hippo, and Hedgehog. Genes from these pathways were highlighted in Table 4 and in the volcano plots shown in Figure 4. Up-regulation of MAPK ligand *HB-EGF* was found at 8 and 12 h, followed by up-regulation of *Ascl1* at 24 and 48 h and *Lin28a* at 48 h. *HB-EGF* is up-regulated at the injury site in Zebrafish, leading to activation of *Ascl1* followed by *Lin28a* which stimulates the formation of multipotent Müller derived progenitor cells in the retina (Wan et al., 2012; Wan and Goldman, 2016). *HB-EGF* activation leads to *Wnt/*β*-Catenin* signaling in zebrafish, the pathway which allows for cell proliferation in the Zebrafish retina (Gallina et al., 2016; Wan and Goldman, 2016). *HB-EGF* is also known to act upstream of β*-Catenin* signaling (Wan et al., 2012). The β*-catenin/Wnt* pathway is essential in the production of Müller-derived progenitor cells in chick (Gallina et al., 2016) and in eye development (Fujimura, 2016). *Bcat1* is up-regulated at 8 and 12 h according to sequencing data, as well as 24 and 48 h in qRT-PCR data. *Dkk3*, an inhibitor of the *Wnt* pathway found in adult mammalian Müller glia (Nakamura et al., 2007), is down-regulated at both 8 and 12 h in our data. *Fzd9* is a *Wnt* receptor known to be activated in the *HB-EGF/Ascl1* pathway by *Wnt* ligands (Ramachandran and Zhao, 2011; Gallina et al., 2016) and is up-regulated at 12 h with qRT-PCR confirmation. *Wnt* signaling occurs in the development of retinal progenitor cells (Meyers et al., 2012). We see down-regulation at 8 and 12 h of markers associated with fully differentiated Müller glia (Todd et al., 2017) (*Bmp4*), as well as inhibitors of cell cycle re-entry (*Hes4, Hes6, Gli3, Let-7*). Fibroblast growth factors are associated with proliferation following injury in the zebrafish retina (McCabe et al., 2006). *FGF9* and *FGF11* are released by retinal cells in *Xenopus* to initiate dedifferentiation and reprogramming (Fukui and Henry, 2012; Meyers et al., 2012). FGFR signaling also promotes retinal regeneration in chick and zebrafish (Fujimura, 2016; Gallina et al., 2016). *Let-7* microRNA, a repressor of retinal regeneration-associated genes in zebrafish such as *Ascl1*, *oct4* and *myc*, has been shown to be inhibited during retinal regeneration in zebrafish (Ramachandran et al., 2010). *Let-7* is a regulator of sonic hedgehog (Shh) signaling (Locker et al., 2006) and is a marker for differentiation from retinal progenitor to adult cell in the developing retina (Xia and Ahmad, 2016). Shh component *Mmp9* is found in injured retinal cells prior to progenitor cell markers and regulates *Ascl1*. Shh component *Gli3* is a negative regulator of *Ascl1* and is reduced in Müller glia progenitor cells (Kaur et al., 2018). Under normal physiological conditions, *Gli3* physically interacts with the *Ascl1* promotor to inhibit its expression (Kaur et al., 2018). Down-regulation of this gene allows the *Ascl1* pathway to move forward and Müller glia to dedifferentiate into multipotent progenitor cells. These signal transduction genes are consistent with regeneration models in other vertebrates and suggest that the same genes and pathways found in animals that can regenerate can lead to dedifferentiation in adult mammals if they are activated.

qRT-PCR was able to verify expression and temporal trends of genes identified through RNA-seq. RNA-seq is highly accurate but represents *n* = 1. By validating with qRT-PCR, we were able to produce three biological replicates of 11 genes that verify the trend in expression seen with RNA-seq. This increased our sample size to *n* = 4 and provided evidence pertaining to the accuracy of results obtained in the RNA-seq data set.

The use of immortalized cell lines in this study could be viewed as a limitation for these studies. Immortalization is by definition an alteration of the genome of cells away from normal physiological conditions. However, results were normalized to the control condition. Therefore, all changes in gene expression were relative to the control in both cell lines. A standard quantity of RNA was used as well to normalize the comparative responses. Future studies will repeat these experiments in primary RPE and Müller glial cells to validate these findings.

Another limitation of this study is that only gene expression up to 48 h was analyzed as experiments were designed to look at early gene expression that led to MGPCs. In future experiments, it will be important to look at the RNA seq data for expression of the progenitor markers beyond 48 h. This data is absent or missing in the current study.

IHC experiments analyzing activated RPE treated Müller glia demonstrated that the conclusion of transcriptomics changes produce cells with a RPC-like fate. Previous studies in Zebrafish have demonstrated many pathways involved in Müller glial dedifferentiation and retinal regeneration that also appear to be involved in PNU-282987-induced dedifferentiation of Müller glia in adult mice. Given that Müller glia proliferate before production of MDPCs in other models, it is expected that progenitor cell markers would appear at later timepoints. At 48 h post-treatment in culture, we see Nestin, VSX2, and OTX2 expression. This provides evidence that these conditions can produce progenitor cell fate in culture. In mammals, damage typically induces proliferation of Müller glia in the form of reactive gliosis, which is not a beneficial outcome as no new neurons are generated. However, this study demonstrates the unique finding that PNU-282987 is able to generate MDPCs in adult mammals in the absence of injury, an outcome with more functional significance to the animals.

Given the data presented above, it was hypothesized that the sDEGs identified in this study are involved pathways of dedifferentiation within the adult mammalian retina following exposure to PNU-282987. The consistency of the data with injury-induced regeneration of the zebrafish retina points to the possibility that the mechanism may be partially conserved. Much work is left to be done to develop a defined pathway of proliferation to MDPCs in adult mammals in response to PNU-282987. However, this study provides a frame of reference to more fully understand the mechanism by which this phenomenon has been shown to occur.

### Implications

The regenerative response to injury in the retina is common in lower vertebrates, such as teleost fish and chick (Stenkamp, 2015). Currently, the adult mammalian neuronal retina is not thought to, under normal physiological conditions, have the ability to restore loss of function caused by neuronal cell death past early development. However, this lab has found a robust neurogenic response in the adult mammalian retina after eye drop application of PNU-282987 (Webster et al., 2017, 2019) without inducing injury. Other research in this area has been met with success at a much more limited scale and requires external manipulations to generate few retinal precursors limited to a specific fate (Yao et al., 2018). The genetic profile and IHC of the MD generated as a result of PNU-282987 application indicate that multiple types of neurons can be generated. Also, these results demonstrate that the pathways involved in adult mice Müller glia dedifferentiation have many similarities with the regenerative capabilities of Müller glia in zebrafish. However, molecules released from PNU-282987 treated RPE cells can stimulate these pathways in adult mammals and bypass typical inhibition of mammalian Müller glia. The results from this study suggest that the inability of adult mammals to induce neurogenesis is not due to unique signaling cascades or genes in the mammalian system. Instead, it is possible that the mammalian Müller glia lack the signaling required to initiate cell division and should direct future research in this field. Ultimately, examining the mechanism by which this occurs will be useful in understanding which factors limit the mammalian system from regeneration in adulthood and how this can be manipulated to treat neurodegenerative diseases of various body systems, including the retina.

The mechanism proposed as a result of this study could lead to the ability to control dedifferentiation in an effort to restore vision in retinal neurodegenerative disease models. At present, PNU-282987’s greatest effects would be in the retina, as it has been reported to inhibit a potassium channel in the heart if given systemically (Duris et al., 2011; Deng et al., 2019). However, understanding the mechanism of PNU-282987’s effect in the retina could allow for manipulation of these pathways to bring about a regenerative response. Ultimately, it is crucial to address the function of the retina after α7 nAChR agonist treatment. Even if new adult retinal neurons are generated after PNU-282987 treatment, one must ask if new cells make appropriate functional connections? Do they make synapses and function to affect visual processing? Can regeneration of new neurons reverse the functional effects of retinal disease, injury or age? One way to address these issues is to analyze electroretinogram (ERG) activity in the rodent eye before and after α7 nAChR agonist treatment with and without injury. These studies are currently underway.

The use of specific α7 nAChR agonists could potentially lead to important clinical treatments for other neurodegenerative diseases in the eye as well as in the brain where α7 nACh receptors have been shown to be involved; such as Alzheimer’s, Parkinson’s and Huntington’s diseases (Foucault-Fruchard and Antier, 2017). α7 nAChRs within the subventricular zone (SVZ), lining the lateral ventricle of the brain, have been found to be involved in neurogenesis in the SVZ when activated (Mudo et al., 2007; Narla et al., 2013; Shabani et al., 2020). As a result, understanding of the mechanism associated with cell cycle reentry of Müller glia in the retina after PNU-282987 treatment is crucial. Understanding the genes and signaling pathways that are involved in this dedifferentiation response may allow future treatments that directly stimulate proliferative pathways or genes without needing PNU-282987. For instance, the pathways identified in this study are known to act in similar ways in other areas of the mammalian central nervous system. *HB-EGF* is widely expressed in the brain, including the hippocampus and cerebral cortex, and is considered to multiple roles in the developing nervous system (Oyagi and Hara, 2012). Canonical *Wnt/*β*-catenin* signaling plays a big role in in tissue patterning in regulating rostral-caudal and medial-lateral patterning in the developing cortex (Noelanders and Vleminckx, 2017). Flushing out the mechanism by which neurogenesis is induced in the retina using PNU-282987 could allow for an understanding of inducible neurogenesis in adult mammals in the CNS systems.

## Data Availability Statement

The datasets generated for this study can be found in the NCBI GEO accessions GSE151477.

## Ethics Statement

The animal study was reviewed and approved by IACUC Western Michigan University.

## Author Contributions

MS was responsible for generating all the data and figures for this study. SW was responsible for helping with analysis and intellectual qPCR and bioinformatic input as well as assisting with generating figures and editing writing. MW was responsible for helping to outline the project and for bioinformatic analysis. CL was the manager for this project and assisted with editing of the writing and analysis. All authors contributed to the article and approved the submitted version.

## Conflict of Interest

The authors declare that the research was conducted in the absence of any commercial or financial relationships that could be construed as a potential conflict of interest.

## Supplementary Material

TABLE S1 Summary of efficiency curves of all primer/probes used in qRT-PCR. All RNA Integrity Number (RIN) values for RNA used were greater than 6.0 and all efficiencies for primer/probes were between 90 and 110%.
