# Supplementary material for: Involvement of HB-EGF/Ascl1/Lin28a Genes in Dedifferentiation of Adult Mammalian Müller Glia
Source: Front Mol Biosci. 2020 Aug 14;7:200. doi: 10.3389/fmolb.2020.00200 (PMC7457012; doi:10.3389/fmolb.2020.00200)
Supplement: TABLE S1 — Summary of efficiency curves of all primer/probes used in qRT-PCR. All RNA Integrity Number (RIN) values for RNA used were greater than 6.0 and all efficiencies for primer/probes were between 90 and 110%. [file Table_1.pdf]

Supplemental Table 1. Primer/Probe Efficiencies	
**Primer/Probe**	**Efficiency Calculation (%)**
HB-EGF	99.13
Ascl1	104.26
Lin28a	97.29
Fgf11	97.77
Fgf9	100.06
Sox9	90.68
Fzd9	105.40
Mmp9	105.92
Gli3	108.53
Bcat1	103.92
Bmp4	104.61
Gapdh	105.02
**Sample**	**RIN**
DMSO Ctrl	8.7
8 Hour	8.6
12 Hour	6.1
24 Hour	6.8
48 Hour	6.1

Supplemental Table 1. Summary of efficiency curves of all primer/probes used in qRT-PCR. All RNA Integrity Number (RIN) values for RNA used were greater than 6.0 and all efficiencies for primer/probes were between 90%-110%.
